# Extracellular Vesicles Derived from Senescent Fibroblasts Attenuate the Dermal Effect on Keratinocyte Differentiation

**DOI:** 10.3390/ijms21031022

**Published:** 2020-02-04

**Authors:** Eun-Jeong Choi, In Sup Kil, Eun-Gyung Cho

**Affiliations:** Basic Research & Innovation Division, R & D Unit, AmorePacific Corporation, Yongin 17074, Korea; ejchoi@amorepacific.com (E.-J.C.); iskil@amorepacific.com (I.S.K.)

**Keywords:** senescence, human dermal fibroblast, extracellular vesicle, exosome, lysosomal activity, epidermal homeostasis

## Abstract

The skin is a multilayered and primary defensive organ. Intimate intercellular communication in the skin is necessary to ensure effective surveillance. Extracellular vesicles (EVs) are being explored for their involvement in intercellular skin communication. The aim of this study was to evaluate how human dermal fibroblasts (HDFs) accelerate EV production during senescence and the effects of senescence-associated EVs on epidermal homeostasis. Replicative senescent HDFs were assessed with senescence-associated β-galactosidase staining and the expression of senescence-related markers. Isolated EVs were characterized by dynamic light scattering and EV marker expression. EVs secreted from untreated young or senescent HDFs, or from those treated with a nSMase inhibitor, antioxidant, and lysosomal activity regulators, were determined by sandwich ELISA for CD81. Human epidermal keratinocytes were treated with young- and senescent HDF-derived EVs. Compared to young HDFs, senescent HDFs produced relatively high levels of EVs due to the increased nSMase activity, oxidative stress, and altered lysosomal activity. The nSMase inhibitor, antioxidant, and agents that recovered lysosomal activity reduced EV secretion in senescent HDFs. Relative to young HDF-derived EVs, senescent HDF-derived EVs were less supportive in keratinocyte differentiation and barrier function but increased proinflammatory cytokine IL-6 levels. Our study suggests that dermis-derived EVs may regulate epidermal homeostasis by reflecting cellular status, which provides insight as to how the dermis communicates with the epidermis and influences skin senescence.

## 1. Introduction

Skin is a primary defensive organ and comprises various cell types that intercommunicate to make it an effective barrier [1]. Upon pathogen exposure, skin cells form cytokine cascades from the epidermis to the dermis, activate vascular endothelial cells, and recruit circulating immune cells. As intercellular mediators, skin cells transmit signals either by direct contact or by releasing soluble factors including proteins and genetic material such as miRNAs, and extracellular vesicles (EVs) transfer biologically active molecules to adjacent and distant cells for communication and intra- and inter-organ regulation [2].

EVs, lipid bilayer-enclosed spherical vesicles ~30–1000 nm in diameter, are either constitutively or actively produced by most organisms and harbor various biomolecules originating from parent cells, which they deliver to neighboring cells [3,4,5]. Based on their mechanism of biogenesis, EVs are classified as exosomes and microvesicles. Exosomes are generated by the fusion of multivesicular bodies (MVBs) with plasma membranes and microvesicles are formed from plasma membranes by evagination and fission [6]. EVs reportedly regulate skin pathophysiology including development, pigmentation, and diseases such as cancer and scleroderma [7,8,9,10]. While adipose-, endothelial cell- or mesenchymal stem cell-derived EVs influence wound healing, pathogen-derived EVs are involved in the pathogenesis of skin disorders such as acne vulgaris and atopic dermatitis [7,11,12].

Skin aging is closely associated with changes in dermal fibroblasts. Aging fibroblasts have senescence-associated secretory phenotypes (SASP), such as the upregulation and enhanced expression of inflammatory cytokines, growth factors, and extracellular matrix-associated factors [13,14]. SASP causes senescent dermal fibroblasts to affect neighboring microvascular endothelial cells, epidermal melanocytes, and keratinocytes [15]. They may alter keratinocyte proliferation, differentiation, and stress responses [16]. Senescence-associated increase in EV secretion is a general feature of aging in various cell types. EVs participate in the senescence, survival, and differentiation of recipient cells [17,18]. Intercellular interactions of senescent fibroblast-derived SASP have been widely investigated. However, the effects of senescent fibroblast-derived EVs on epidermal homeostasis remain to be elucidated.

Here, we evaluated increases in EV production and secretion in senescent human dermal fibroblasts (HDFs) as one of the senescence phenotypes and the effects of senescent HDF-derived EVs on epidermal keratinocytes. Senescent HDFs produce more EVs than young HDFs because the former have elevated neutral sphingomyelinase (nSMase) activity and altered lysosomal activity. Senescent HDF-derived EVs are less effective at supporting keratinocyte differentiation and barrier function than young HDF-derived EVs. Therefore, dermal EVs may be important regulatory factors in epidermal homeostasis and senescence-associated EVs may be targets for suppression in skin homeostasis maintenance.

## 2. Results

### 2.1. Increased Extracellular Vesicle Secretion in Replicative Senescent Dermal Fibroblasts

**Figure 1:**
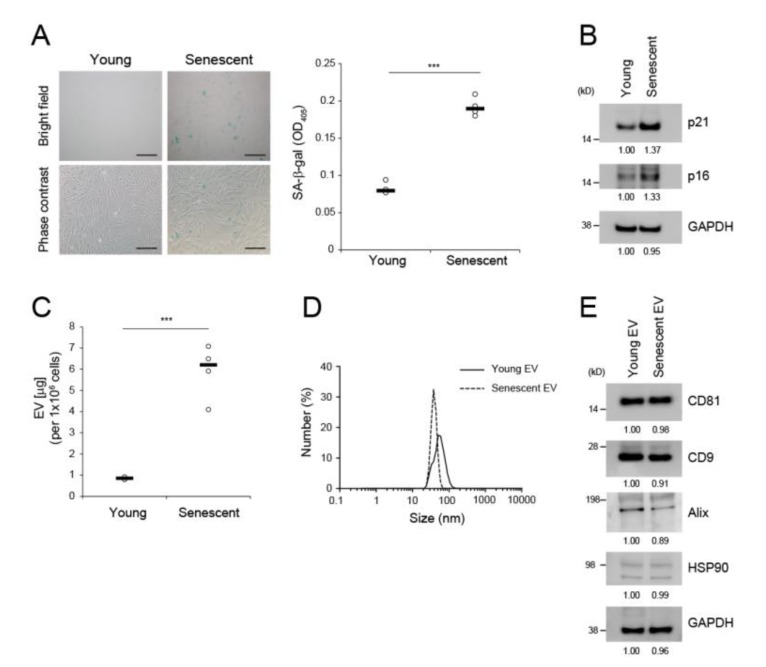
EV secretion was increased in replicative senescent dermal fibroblasts. (**A**) Senescence-associated (SA)-β-gal assay in young HDFs (PDL < 10) and senescent HDFs (PDL > 50). Scale bar = 50 μm. Data are means ± SD of three independent experiments on two independent senescent cell lines (*** *p* < 0.001). (**B**) Western blot analysis of p21 and p16 in young versus senescent HDFs. GAPDH was the loading control. (**C**) Levels of EVs derived from equal numbers of young and senescent HDFs. Protein concentrations in isolated EVs were determined by BCA assay. Data are means ± SD of four independent experiments using two independent senescent cell lines (*** *p* < 0.001). (**D**) Dynamic light scattering analysis of EVs derived from young and senescent HDFs. (**E**) Western blot analyses of EV markers. Five micrograms of EV protein were subjected to immunoblot analysis with anti-CD81, anti-CD9, anti-Alix, and anti-HSP90. GAPDH was the loading control.

HDFs were derived primarily from adult human skin. Their growth was maintained until the population doubling level (PDL) 10 for young HDFs. We established replicative senescent HDFs by continuously growing and sub-culturing the cells > PDL 50. By that time, cells were senescent and had elevated senescence-associated β-galactosidase activity (Figure 1A) and increased expression of the senescence markers p21 and p16 (Figure 1B). Cells at this stage also showed upregulated expression of factors associated with advanced (IL-8, NF-κB) or very advanced (MMP1) HDF senescence (Supplementary Figure S1) [19]. We isolated EVs from 10^6^ young (PDL < 10) and 10^6^ senescent (PDL > 50) HDFs by conventional ultracentrifugation [8]. Senescent HDFs produced considerably more EVs (6.49 ± 0.83-fold) than young HDFs based on protein concentration (Figure 1C). Dynamic light scattering analysis revealed that young and senescent HDF-derived EVs were 20–500 nm in diameter with averages of 160.4 ± 3.3 nm and 123.0 ± 8.2 nm, respectively (Figure 1D). Both EVs expressed the markers CD81, CD9, Alix, and HSP90 at similar levels when equal amounts of EV proteins were loaded (Figure 1E). EV production also increased under UV irradiation and in adult HDFs relative to that in unirradiated and neonatal HDFs, respectively (Supplementary Figure S2). Therefore, senescent HDFs produce more typical EVs than young HDFs.

### 2.2. Increased EV Biogenesis in Senescent Dermal Fibroblasts

**Figure 2:**
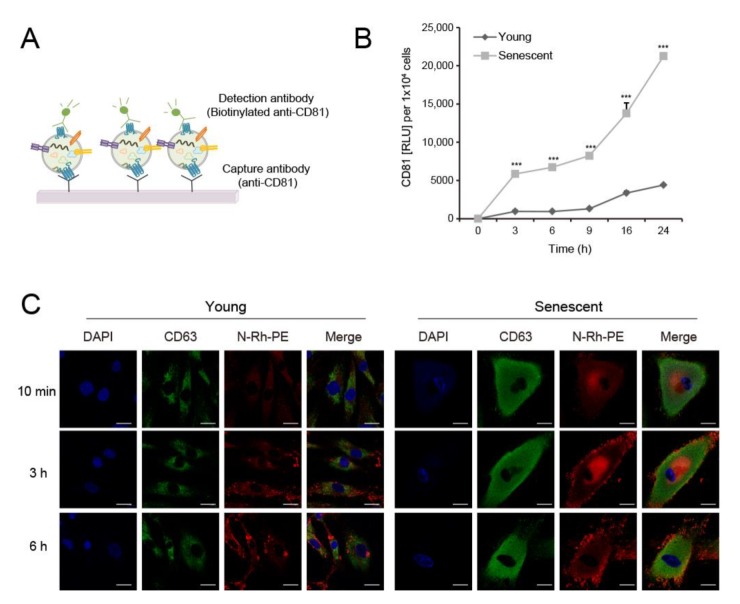
EV biogenesis increased in senescent dermal fibroblasts. (**A**) Schematic image of sandwich ELISA using anti-CD81 antibody to detect EVs. (**B**) Conditioned media used to culture equal numbers of young and senescent HDFs were harvested at the indicated time points. EV levels were quantitated by sandwich ELISA for CD81. Data are means ± SD of three independent experiments using a senescent cell line (*** *p* < 0.001). (**C**) HDFs were labeled with a fluorescent lipid molecule N-Rh-PE (red) for the indicated time periods and co-stained with anti-CD63 antibody (green). Nuclei were stained with 4′6-diamidino-2-phenylindole (DAPI; blue). Fluorescence images were taken under a confocal microscope. Representative images are shown. Scale bar = 20 μm.

CD81 is highly enriched in EVs and frequently used to verify them and exosomes [20]. We used anti-CD81 antibody to develop a sandwich ELISA for CD81(+)-EV detection in the culture media, by which comparative changes in released EV levels in response to certain conditions can be evaluated (Figure 2A). The sandwich ELISA system successfully compared EV production kinetics in young and senescent HDFs. The amounts of EVs produced during the same time period were remarkably higher in senescent than in young HDFs (Figure 2B). EV secretion before 3 h was much stronger in senescent than in young HDFs (Figure 2B). To determine whether this difference was caused by EV biogenesis, we labeled cells with the fluorescent lipid dye N−Rh−PE, which accumulates in multivesicular bodies (MVBs) or multivesicular endosomes (MVEs). We measured fluorescence signals over time. After labeling for 3 h, N-Rh-PE was concentrated in the perinuclear region and plasma membrane of young and senescent HDFs (Figure 2C). Nevertheless, the fluorescence intensity per cell was higher in senescent- than in young HDFs. CD63, a marker for MVB and lysosomes, markedly increased in aged cells and tissues [21]. CD63 was highly expressed in senescent HDFs (Figure 2C). Considering that N-Rh-PE and CD63 intracellular localization patterns were similar for young and senescent HDFs but their fluorescence intensities differed, compared to young cells, senescent HDFs may produce considerable amounts of EVs because of their large numbers of MVBs.

### 2.3. Enhanced nSMase Activity in Senescent HDFs Increases EV Production, which is Reversed by Antioxidants

**Figure 3:**
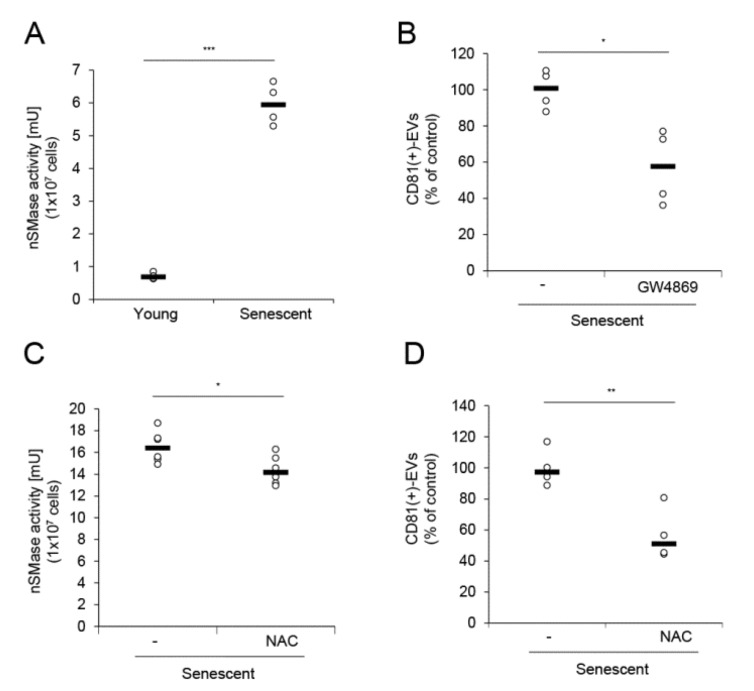
Enhanced nSMase activity in senescent HDFs increased EV production. (**A**) nSMase activity was determined for equal numbers of young and senescent HDFs with the nSMase activity assay kit. (**B**) Senescent HDFs were treated with vehicle (-) or GW4869 (5 μM) for 24 h. Relative EV quantity was analyzed by sandwich ELISA for CD81 in each conditioned medium. **C**–**D** Equal numbers of senescent HDFs were treated with vehicle (-) or *N*-acetyl cysteine (NAC; 5 mM) for 24 h and nSMase activity was measured (**C**). Senescent HDFs were treated with NAC and the relative EV quantity was measured by sandwich ELISA for CD81 in each conditioned medium (**D**). Data are means ± SD of three independent experiments using a senescent cell line (* *p* < 0.05; ** *p* < 0.01; *** *p* < 0.001).

Formation of intraluminal vesicles (ILVs) within MVBs is associated with the secretion of exosomes and regulated by neutral sphingomyelinase (nSMase) [3,22]; therefore, the activity of this enzyme is associated with the number of exosomes produced [23]. We measured nSMase activity in young and senescent HDFs. Consistent with a previous report stating that nSMase activity is elevated in senescent cells [24], senescent HDFs showed considerably upregulated nSMase activity compared to young HDFs (Figure 3A). When senescent HDFs were treated with the nSMase inhibitor GW4869, EV production was significantly decreased (Figure 3B). Therefore, elevated nSMase activity partially accounts for increased EV production in senescent HDFs. Reactive oxygen species (ROS) levels increase in response to functional deterioration of senescent cell mitochondria [25,26]. nSMase is activated by ROS and can be inhibited by antioxidants [27,28]. We examined whether the antioxidant N-acetyl-cysteine (NAC) inhibits nSMase and, by extension, reduces EV production. NAC treatment of senescent HDFs significantly reduced nSMase activity, which was accompanied by decreased EV production (Figure 3C,D). Taken together, enhanced nSMase activity, which may partly result from increased ROS levels in senescent cells, increases EV production; therefore, various antioxidants can be used to mitigate EV production as well as cellular ROS.

### 2.4. Dysfunctional Lysosomal Activity in Senescent HDFs is Related to Increased EV Secretion

**Figure 4:**
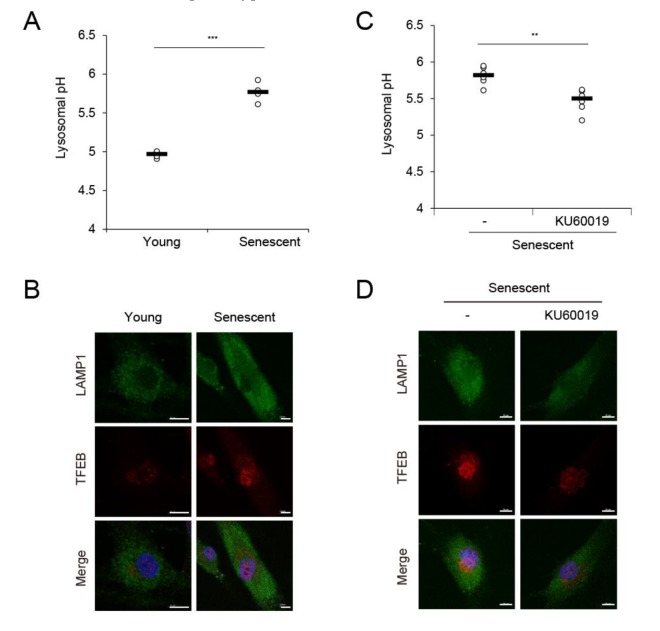

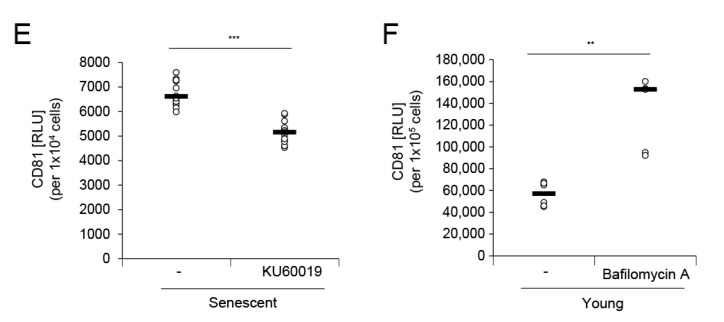
Dysfunctional lysosomal activity in senescent HDFs increased EV secretion. (**A**) Lysosomal pH in young and senescent HDFs was measured with a LysoSensor Yellow/Blue DND-160. (**B**) Representative confocal images for LAMP1 (green) and TFEB (red) in young and senescent HDFs. Nuclei were stained with DAPI (blue). Scale bar = 10 μm. (**C**) Senescent HDFs were treated with vehicle (-) or KU60019 (0.5 μM) for 25 d and lysosomal pH was measured. (**D**) Confocal images for LAMP1 (green) and TFEB (red) in senescent HDFs either untreated or treated with KU60019. Nuclei were stained with DAPI (blue). Scale bar = 10 μm. (**E**) Senescent HDFs were treated with vehicle (-) or KU60019 (0.5 μM) for 25 d and relative EV quantity was measured by sandwich ELISA for CD81 in each conditioned medium. (**F**) Young HDFs were treated with vehicle (-) or bafilomycin A (100 nM) for 24 h and relative EV quantity was measured by sandwich ELISA for CD81 in each conditioned medium. Data (in **A**,**C**,**E**,**F**) are means ± SD of three independent experiments using a senescent cell line (** *p* < 0.01; *** *p* < 0.001).

MVBs are routed to either the plasma membrane or the lysosome, which results in ILV secretion as exosomes or their lysosomal degradation, respectively [3,26,29]. We hypothesized that lysosomal activity is related to the degree of exosome secretion. In senescent cells, diminished lysosomal activity enhances a feedback loop between lysosomes and mitochondria by accumulating ROS [26] which, in turn, may affect nSMase activity. We measured lysosomal pH, one indicator of lysosome functionality, in young and senescent HDFs and found that it was higher in senescent than in young HDFs (Figure 4A). Senescent HDFs upregulated the lysosomal marker LAMP1 and the transcription factor TFEB, which controls lysosome biogenesis (Figure 4B). Elevation of these proteins reflects a marked increase in lysosomal mass but diminished lysosomal activity [26,30]. KU60019, an ataxia telangiectasia mutated (ATM) inhibitor, recovers lysosome function by restoring ATM-blocked dimerization of ATPase subunits and alleviates senescence [31]. When senescent HDFs were treated with various KU60019 concentrations for 25 d, SA-β-gal activity decreased in a dose-dependent manner and p16 and p21 were significantly downregulated (Supplementary Figure S3A,B). At the same time, nSMase activity was significantly reduced at the same protein concentrations or in the presence of equal numbers of senescent HDFs (Supplementary Figure S3C). Moreover, KU60019 lowered lysosomal pH and downregulated intracellular LAMP1 and TFEB (Figure 4C,D; Supplementary Figure S3B). As a result, it restored lysosome function in senescent HDFs and, by extension, lowered EV secretion in senescent HDFs (Figure 4E). To investigate whether lysosomal activity is directly associated with EV secretion, we treated young HDFs with the lysosome acidification inhibitor bafilomycin A [32] and compared EV levels before and after treatment. Bafilomycin A increased EV secretion in young HDFs to levels comparable with those observed in senescent HDFs (Supplementary Figure S4). Therefore, dysfunctional lysosomal activity enhances EV secretion in senescent cells, and agents recovering lysosomal activity may lower EV secretion and alleviate senescence-related phenotypes.

### 2.5. Senescent Fibroblast-Derived EVs Attenuate the Dermal Effect on Keratinocyte Differentiation but Evoke Proinflammatory Cytokine IL-6

**Figure 5:**
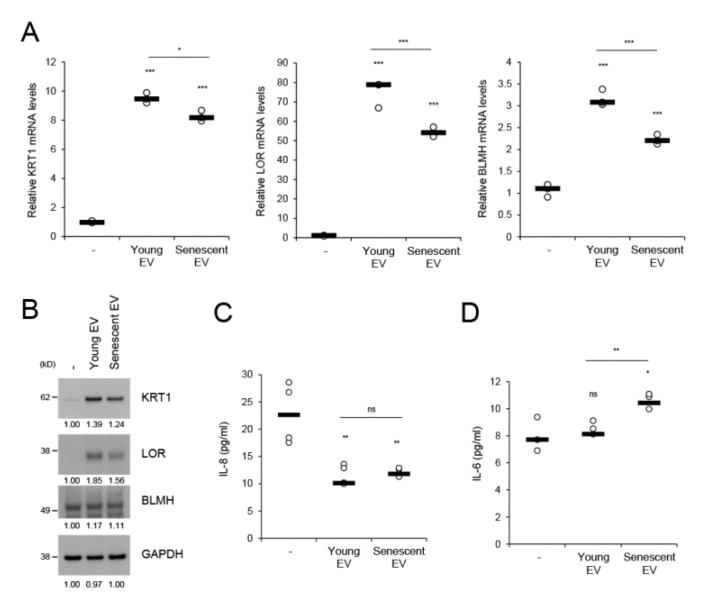
Senescent HDF-derived EVs attenuate the dermal effect on keratinocyte differentiation but evoke proinflammatory cytokine IL-6. Human epidermal keratinocytes (HEKs) in culture were treated with EVs derived from 5 × 10^5^ young and senescent HDFs for 4 days. HEKs cultured without EV treatment for 4 d served as the control (-). Cells were analyzed for mRNA (**A**) and protein (**B**) expression of keratinocyte differentiation-related (KRT1, LOR) or barrier function-related (BLMH) markers by qRT-PCR and western blot analyses, respectively. The mRNA levels were normalized to those of RPL13A. GAPDH was the loading control for western blot analysis. KRT1, keratin 1; LOR, loricrin; BLMH, bleomycin hydrolase. (**C**,**D**) HEKs were treated with EVs derived from 5 × 10^5^ young and senescent HDFs. Conditioned media at 24 h and 4 d post-treatment with EVs were harvested for the determination of secreted IL-8 (**C**) and IL-6 (**D**) levels, respectively, with specific ELISA kits. Data (in **A**,**C**,**D**) are means ± SD of three independent experiments using two senescent cell lines (* *p* < 0.05; ** *p* < 0.01; *** *p* < 0.001; ns, not significant).

EVs facilitate intercellular communication and enable cells to exchange proteins, lipids, and nucleic acids [22]. We investigated whether senescent HDF-derived EVs affect homeostatic regulation of keratinocytes. During in vitro culture from day 1 to 4, HEKs differentially expressed the epidermal differentiation-related markers (KRT1, LOR, and BLMH), cell cycle inhibitors (p16 and p21), and cytokines (IL-6 and IL-8) (Supplementary Figure S5). BLMH is a filaggrin cleavage enzyme that generates natural moisturizing factors and whose levels are reduced in dry skin and atopic dermatitis [33,34]. When HEKs were treated for 4 d with EVs derived from equal numbers of young or senescent HDFs, KRT1, LOR, and BLMH mRNA expression levels were markedly increased by young HDF-derived EVs compared to mock-treatment. However, these effects were significantly diminished by senescent HDF-derived EVs (Figure 5A). These differential effects driven by young and senescent HDF-derived EVs were reflected in the protein levels of each marker (Figure 5B) and reproduced when both EVs were applied to keratinocytes at the same protein concentrations (Supplementary Figure S6A). mRNA expression of p63, a master transcription factor for epidermal proliferation and differentiation, was reduced by high concentrations of senescent HDF-derived EVs, but not by young HDF-derived EVs (Supplementary Figure S6B). However, young and senescent HDF-derived EVs did not significantly differ in terms of the induction of cellular senescence markers such as p16 and p21 (Supplementary Figure S6C). We measured IL-6 and IL-8 levels upon dermal EV application to keratinocytes. IL-8 was significantly downregulated by both dermal EVs, whereas IL-6 was upregulated in response to senescent HDF-derived EVs (Figure 5C,D). Therefore, IL-6 may participate in senescent EV-associated epidermal regulation. These results suggest that dermal fibroblast-derived EVs play crucial roles in homeostatic keratinocyte regulation, namely, cellular differentiation and moisturizing factor generation. Nevertheless, fibroblast senescence attenuates this EV-mediated intercellular effect.

## 3. Discussion

In the human skin, the dermal effect in epidermal homeostasis regulation has not been well verified. Therefore, this study aimed to provide insight into how dermal EVs reflecting cellular status, such as young vs. senescent, influence neighboring or remote (epidermal) cells and participate in skin homeostasis regulation. We evaluated the relative increase in EV secretion in senescent HDFs, the less supportive effect of senescent HDF-derived EVs on keratinocyte differentiation, and the relative increase in inflammatory cytokine IL-6 caused by senescent HDF-derived EVs.

To generate dermal EVs reflecting cellular senescent status, we established replicative senescent HDFs at PDL > 50. Consistent with a previous report stating that approximately 20% of all cells at ~PDL 75 presented low levels of SA-β-gal activity [19], a subset of the cell population was SA-β-gal-positive and its expression was heterogeneous. Therefore, senescent cell populations require a long time to attain homogeneity and strong SA-β-gal activity. We predicted that ≥ 20% of our SA-β-gal-positive cell population would be at PDL > 50 in view of the heterogeneous staining and the enzyme activity of SA-β-gal (Figure 1A). Assays of cell cycle inhibitors, SA-β-gal activity, SASP-related cytokines, signaling molecules, and MMPs/TIMPs and cell cycle analysis may be necessary to confirm cellular senescence.

Senescence-associated increases in EV secretion may be a general feature of cellular senescence and have been demonstrated in fibroblasts, epithelial cells, and cancer cells [18,35,36,37]. This increase is part of SASP, and elevated p53 during senescence increases EV secretion [18,37]. p53 upregulates several exosome-related genes such as TSAP6, caveolin-1, the ESCRT-III subunit Chmp4, and Rab5B/Rab27B, thereby enhancing EV production or secretion [38,39]. In our study, however, the p53 inhibitor pifithrin-α did not reduce EV secretion from senescent HDFs (Supplementary Figure S7). Therefore, the p53 pathway may not be directly associated with EV secretion at least in replicative senescent HDFs. As EV secretion increased in UV-irradiated HDFs (Supplementary Figure S2A), p53 could regulate EV production under UVR conditions as it is a key factor in DNA damage-induced cellular phenomena. Thus, various signaling pathways may regulate EV production under different cellular conditions.

Dysfunctional lysosomal activity has also been proposed as an EV secretion mechanism [37]. ILVs that accumulate in MVB lumens are distributed to either the plasma membrane or lysosomes. One pathway is promoted by inhibition of the other because of the limited amount of intracellular MVBs [37]. If a cell has dysfunctional lysosomes, MVBs are more likely to fuse with the plasma membrane than lysosomes, so that more EVs are secreted from cells. Lysosome defect-associated EV secretion could compensate for lysosomal dysfunction or overload to dispose misfolded proteins. Lysosome inhibition with bafilomycin A in young HDFs (Figure 4F), the lysosomotropic agent chloroquine, and mutation of the genes regulating lysosomal functionality all increase EV secretion [37,40,41]. Therefore, senescent HDFs may increase EV secretion via dysfunctional lysosomal activity rather than an elevated p53 pathway. Moreover, nSMase activity was reduced when KU60019 recovered lysosomal activity (Supplementary Figure S3C). The role of this enzyme may be related to EV secretion associated with lysosome defects. The latter raises ROS levels which, in turn, activate nSMase and, by extension, generate more EVs.

When lysosomal activity was recovered by KU60019, an inhibitor of ATM, EV secretion was reduced in replicative senescent HDFs (Figure 4). Under these senescence conditions, ATM may regulate lysosomal functionality rather than genomic instability. ATM inhibition may also prevent pre-senescent cells from becoming senescent by lowering lysosomal pH. Besides, KU60019 may regulate ATM activity under genotoxic stress-induced senescence conditions, which occur in response to γ-irradiation and UVR and activate γ-H2AX foci [42,43]. Instead of reduced lysosome activity in senescent cells, EV secretion may remove misfolded or abnormal proteins. However, emerging roles for EVs were demonstrated in cellular senescence, survival, and differentiation [35,36,44,45,46]; therefore, senescent HDF-derived EVs may be essential in regulating age-related skin disorders including abnormal epidermal differentiation and chronic inflammation.

Regarding the impact of EVs on skin homeostasis, keratinocyte-derived EVs enhance melanin synthesis by upregulating melanosomal protein [9]. Conversely, melanocyte-derived EVs protect melanocytes and keratinocytes from UV irradiation in an autocrine or paracrine manner [8,10]. *Propionibacterium acnes*- or *Staphylococcus aureus*-derived EVs are associated with acne vulgaris and atopic dermatitis, respectively [11,12]. Cellular senescence or aging is the progressive loss of the ability to maintain homeostasis and is associated with various chronic diseases. While SASPs are released from senescent cells and locally affect skin physiology, senescent cell-derived EVs may remotely propagate senescent phenotypes by transferring senescence-related substances intracellularly at a high concentration or delivering beneficial substances at low doses. Since young or senescent HDF-derived EVs differ in size and distribution (Figure 1D), it may be informative to analyze EV components to determine whether EV composition reflects cellular status and which components mainly get involved in epidermal regulation. Based on the result that senescent HDF-derived EVs did not upregulate p16 or p21 in keratinocytes (Supplementary Figure S6C), these markers may not be directly transferred to recipient cells via EVs or induced by senescent EVs in the short term. However, we do not exclude the possibility that other bioactive molecules, including proteins, lipids, nucleic acids, and metabolites trapped in EVs may be directly involved in this regulation by transmitting the signals intracellularly after being transferred via endocytosis. EVs are transported into recipient cells via several mechanisms, including receptor-mediated endocytosis, direct fusion, phagocytosis, and caveolae- or clathrin-mediated endocytosis [47]. To know which uptake pathways and signaling molecules are involved in EV-evoked keratinocyte responses would be helpful for verifying the role of dermal EVs in maintaining epidermal homeostasis and for developing a way to diminish dermis-originated senescence effects in the human skin.

Keratinocytes migrate from the basal to the cornified layer then detach. This process is accompanied by keratinocyte proliferation and differentiation, which are the two major components of epidermal homeostasis and often disturbed in aged skin and skin disorders. The phenotypes observed in acne lesions such as hyperproliferation, diminished differentiation, and epidermal inflammation are promoted by *P. acnes*-derived EVs (PEVs) relative to parent cells [11]. These PEV-mediated effects on epidermal homeostasis were the opposite of those controlled by dermal fibroblast-derived EVs. The latter supported keratinocyte differentiation and decreased inflammatory IL-8 levels without affecting p63 expression (Figure 5; Supplementary Figure S6B). Therefore, the poor supportive effect of senescent dermal EVs on keratinocyte differentiation compared to that of young EVs may explain the abnormal epithelial differentiation in aged skin and could be another senescence-associated phenotype. The increase in proinflammatory IL-6 resulting in epidermal differentiation attenuation [48] could partially explain the reduction in keratinocyte differentiation after senescent EV treatment. 

Besides keratinocyte differentiation, dermal EVs could mediate effects on matrix construction/remodeling and wound healing. Based on recent studies [45,49], a short-time treatment of stress-induced, senescent fibroblast-derived small EVs accelerated scratch closure of epidermal keratinocytes via vesicular miR-23a-3p, whereas long-term incubation with these EVs impairs keratinocyte differentiation compared to quiescent fibroblast-derived EVs, which is consistent with our result using replicative senescent HDF-derived EVs. It may be possible that young and senescent HDF-derived EVs have variable influence on the wound healing process. However, whether replicative senescent HDF-derived EVs would be more effective than young HDF-derived EVs in wound healing remains to be investigated, because vesicular components will differ according to cellular status (young vs. senescent; stress-induced senescence vs. replicative senescence) and consequently, EV-evoked cellular responses may be different according to vesicular components, showing the necessity of massive analyses for young vs. senescent dermal EVs to verify putative vesicular components involved in pathophysiological regulation in the human skin.

Our results suggested that senescent HDFs secrete lipid bilayer-enclosed EVs in relatively high amounts compared to young HDFs due to elevated nSMase activity, oxidative stress, and dysfunctional lysosomal activity. As senescence-associated dermal EVs are relatively less supportive of epidermal differentiation and barrier function and induce the proinflammatory cytokine IL-6, inhibiting EV release from senescent cells using the associated inhibitors, i.e., GW4869, NAC, and KU60019, and preventing its diffusion to other skin cells and layers may help alleviate senescent skin phenotypes.

## 4. Materials and Methods

### 4.1. Cell Culture and Chemicals

Human dermal fibroblasts derived from the skin of a 33-year old adult (HDFs) or neonatal foreskin (HDFs-Neo) were purchased (Lonza, Basel, Switzerland) and cultured in DMEM supplemented with 10% FBS (Lonza, Basel, Switzerland). Replicative senescence was induced by transferring confluent HDFs to two new dishes, restoring them to confluence, and doubling the population as previously reported [19]. At least, two independent senescent cell lines from two different lots of HDFs were established to study senescence-associated phenotypes. Human epidermal keratinocytes-neonatal (HEKs; Lonza, Basel, Switzerland) were cultured in KBM-GOLD medium supplemented with KGM-Gold BulletKit (Lonza, Basel, Switzerland). GW4869 [50], *N*-acetyl-cysteine, KU-60019, and bafilomycin A were purchased from Sigma-Aldrich Corp. (St. Louis, MO, USA).

### 4.2. Determination of Population Doubling Level (PDL)

HDFs were seeded at 5 × 10^5^ cells in 100-mm culture dishes. The population doubling level (PDL) was calculated based on the following formula:

log2 ((No. cells harvested) / (No. cells seeded)) + previous PDL(1)

The PDL of the cells received from the manufacturer was unknown. Thus, the first PDL after the initial culture was defined as zero [51]. Cells at various PDLs were frozen and stored for subsequent experiments.

### 4.3. Senescence Associated-β-gal (SA-β-gal) Assay

SA-β-gal staining was performed with a β-gal staining kit (Invitrogen, Carlsbad, CA, USA) and SA-β-gal activity of cells (2 × 10^5^) from young and senescent HDFs was determined with a mammalian β-Gal assay kit (Thermo Fisher Scientific, Waltham, MA, USA) according to the manufacturer’s instructions. Detailed protocols are described in the Supplementary Material. 

### 4.4. Isolation of Extracellular Vesicles

HDF-derived EVs were isolated as previously described, with minor modifications [52]. Confluent cells were washed twice with PBS and grown in basal DMEM medium (Lonza, Basel, Switzerland) for 48 h. The supernatants were sequentially centrifuged at 500× *g* for 10 min then 3000× *g* for 20 min at 4 °C to remove cells and debris then ultracentrifuged at 100,000× *g* for 2 h at 4 °C. EV pellets were resuspended in HEPES-buffered saline (HBS).

### 4.5. Dynamic Light-Scattering Analysis

The EVs were measured by dynamic light-scattering with a Zetasizer Nano ZS (Malvern Instruments, Worcestershire, UK). Data are averages of five measurements.

### 4.6. Western Blotting

HDFs or HEKs were lysed in RIPA cell lysis buffer (EMD Millipore, Billerica, MA, USA) containing a protease and phosphatase inhibitor cocktail (Sigma-Aldrich Corp., St. Louis, MO, USA). After centrifugation at 13,000 rpm for 10 min, protein concentration was measured by a BCA assay (Thermo Fisher Scientific, Loughborough, UK) and the supernatant was loaded onto a 5–20% gradient gel for immunological analyses using indicated antibodies. Densitometry analysis of each band intensity was performed using ImageJ software (https://imagej.nih.gov/ij/). Full blot image for each western blot data is included in the Supplementary Figure S8. Detailed protocols for western blotting and antibody information are described in the Supplementary Material.

### 4.7. Sandwich ELISA for EV Detection

Mouse monoclonal anti-CD81 antibody (Abcam; clone No. B1.3.3.22) was immobilized on 96-well microtiter plates (Greiner Bio-one, Frickenhausen, Germany) overnight and blocked with 1% BSA in PBS for 1.5 h. HDFs were treated with vehicle or reagents for 24 h (GW4869, NAC, and bafilomycin A) or for 25 d (KU60019), in which period, cells were replaced with the fresh medium supplemented with KU60019 every three days according to a previous report [31]. The culture supernatants were added to pre-coated 96-well microtiter plates after pre-clearing through centrifugation, for which detailed protocols are described in the Supplementary Material.

### 4.8. Immunocytochemistry

HDFs were grown on 0.1% (*w*/*v*) gelatin-coated coverslips. The cells were incubated with 5 μM N-Rh-PE (Avanti Polar Lipids, Alabaster, AL, USA) for 30 min at 4 °C and washed extensively with cold PBS. The samples were mounted and viewed under a confocal microscope (LSM 700; Carl Zeiss AG, Jena, Germany) under the same exposure conditions. Detailed protocols are described in the Supplementary Material. 

### 4.9. Neutral Sphingomyelinase Activity Assay

Neutral sphingomyelinase activity in young and senescent HDFs was measured with a nSMase assay kit (Echelon Biosciences, Salt Lake City, UT, USA) according to the manufacturer’s instruction.

### 4.10. Measurement of Lysosomal pH

Lysosome pH was measured with a LysoSensor Yellow/Blue DND-160, a dye with two distinct optimal pH sensitivities (Molecular Probes, Eugene, OR, USA) according to the manufacturer’s protocol and previous reports [53,54]. The detailed protocols are described in the Supplementary Material.

### 4.11. EV Treatment

HEKs were seeded in a six-well plate and cultured for 24 h under growth medium conditions. The cells were treated with EVs derived from equal numbers of young (PDL < 10) and senescent (PDL > 55) HDFs. The cells and supernatant were harvested at the indicated time points for western blot or RT-qPCR.

### 4.12. Statistical Analysis

The data were analyzed using the Student’s *t*-test and expressed as the mean ± standard deviation (SD). All experiments were performed at least three times, and representative results are shown.

## Supplementary Materials

Supplementary materials can be found at https://www.mdpi.com/1422-0067/21/3/1022/s1.

## Abbreviations

EVs	Extracellular vesicles
HDFs	Human dermal fibroblasts
MVBs	Multivesicular bodies
SASP	Senescence-associated secretory phenotypes
nSMase	Neutral sphingomyelinase
ILVs	Intraluminal vesicles
ROS	Reactive oxygen species
NAC	N-acetyl-cysteine
ATM	Ataxia telangiectasia mutated
HEKs	Human epidermal keratinocytes
PEVs	*P. acnes*-derived EVs
PDL	Population doubling level
HBS	HEPES-buffered saline
SD	Standard deviation
