# Supplementary material for: Extracellular Vesicles Derived from Senescent Fibroblasts Attenuate the Dermal Effect on Keratinocyte Differentiation
Source: Int J Mol Sci. 2020 Feb 4;21(3):1022. doi: 10.3390/ijms21031022 (PMC7037765; doi:10.3390/ijms21031022)
Supplement: Supplementary file 1 [file ijms-21-01022-s001.pdf]

Supplementary information

Supplementary materials and methods

Senescence associated-β-gal (SA-β-gal) assay

SA-β-gal staining was performed with a β-gal staining kit (Invitrogen, Carlsbad, CA, USA) according to the manufacturer's instructions. Briefly, cells were washed with phosphate-buffered saline (PBS), fixed with fixative solution for 10 min at room temperature, and incubated in the β-gal staining solution for 2 h at 37 °C. SA-β-gal was quantitated with a mammalian β-Gal assay kit (Thermo Fisher Scientific, Waltham, MA, USA). Cells were collected in PBS and extracted with M-PER mammalian protein extraction reagent (Thermo Fisher Scientific, Waltham, MA, USA). After centrifugation at 13,000 rpm for 15 min, 50 μL β-Gal assay reagent was added to 50 μL supernatant. The reaction proceeded for 30 min at 37 °C and absorbance was measured at 405 nm with a Synergy H2 microplate reader (BioTek, Winooski, VT, USA).

Western blotting

Human dermal fibroblasts (HDFs) or human epidermal keratinocytes (HEKs) were lysed in RIPA cell lysis buffer (EMD Millipore, Billerica, MA, USA) containing a protease- and phosphatase inhibitor cocktail (Sigma-Aldrich Corp., St. Louis, MO, USA). The cell lysate was collected by centrifugation at 13,000 rpm for 10 min and the protein concentration was measured by a BCA assay (Thermo Fisher Scientific, Loughborough, UK). Proteins were separated by SDS-PAGE, transferred to PVDF membranes (EMD Millipore, Billerica, MA, USA), and detected by the following antibodies: anti-p21 (Cell Signaling Technology, Beverly, MA, USA), anti-p16 (Cell Signaling Technology), anti-GAPDH (Santa Cruz Biotechnology, Dallas, TX, USA), anti-CD81 (Cat. No. 555675; BD Biosciences, San Jose, CA, USA), anti-CD9 (Cat. No. ab65230; Abcam, Cambridge, MA, USA), anti-Alix (Cat. No. #2171; Cell Signaling Technology), anti-HSP90 (Cat. No. #4874; Cell Signaling Technology), anti-keratin 1 (Biolegend, San Diego, CA, USA), anti-loricrin (Biolegend), and anti-bleomycin hydrolase (Abcam).

Sandwich ELISA for EV detection

Mouse monoclonal anti-CD81 antibody (Abcam; clone No. B1.3.3.22) was immobilized on 96-well microtiter plates (Greiner Bio-one, Frickenhausen, Germany) overnight and blocked with 1% BSA in PBS for 1.5 h. HDFs were treated with vehicle or reagents for 24 h (GW4869, NAC, and bafilomycin A) or 25 d (KU60019). The culture supernatants were pre-cleared by sequential centrifugation at 500 × *g* for 10 min and 3,000 × *g* for 20 min, added to pre-coated 96-well microtiter plates containing anti-CD81 antibody, and incubated for 2 h. They were incubated with biotinylated mouse monoclonal anti-CD81 antibody (BD Biosciences; clone No. JS-81) for another 2 h and streptavidin (R&D Systems, Minneapolis, MN, USA) was added to each well. After 30 min of incubation, luminescence was measured on a Wallace Victor 1420 multilabel plate reader (PerkinElmer, Norwalk, CT, USA). All steps were performed at room temperature.

Immunocytochemistry

HDFs were grown on 0.1% (w/v) gelatin-coated coverslips. The cells were incubated with 5 μM N-Rh-PE (Avanti Polar Lipids, Alabaster, AL, USA) for 30 min at 4 °C and washed extensively with cold PBS. They were then fixed with 4% (v/v) formaldehyde, permeabilized with 0.2% (v/v) Triton X-100 in PBS, blocked with 5% (v/v) horse serum in PBS, and incubated with anti-CD63 (Abcam, Cambridge, UK), anti-LAMP1 (Abcam, Cambridge, UK), or anti-TFEB antibodies (Cell Signaling Technology). After incubating with AlexaFluor 488-conjugated donkey anti-rabbit (Invitrogen, Carlsbad, CA, USA) or AlexaFluor 594-conjugated donkey anti-mouse (Invitrogen, Carlsbad, CA, USA), the samples were mounted with Vectashield mounting medium containing DAPI (Vector

Laboratories, Burlingame, CA, USA) and viewed under a confocal microscope (LSM 700; Carl Zeiss AG, Jena, Germany) under the same exposure conditions.

Measurement of lysosomal pH

Lysosomal pH was measured using LysoSensor Yellow/Blue DND-160 (Molecular Probes, Eugene, OR, USA) according to the manufacturer's protocol[1]. Briefly, NHDFs were collected with trypsin, stained with 1:200 LysoSensor Yellow/Blue DND-160 in standard medium for 10 min, washed with PBS, and suspended in 2-(*N*-morpholino)ethanesulfonic acid (MES) buffer (pH 7.0), and then fluorescence was measured using a TECAN M200 PRO fluorometric plate reader (Tecan, Männedorf, Switzerland) at an excitation wavelength of 365 nm and an emission wavelength of 450 nm for blue fluorescence and an excitation wavelength of 385 nm and an emission wavelength of 550 nm for yellow fluorescence. Standard curves were plotted for each sample with pH-fixed MES buffer (pH 4.0–6.5) containing 10 μM nigericin (Sigma-Aldrich Corp., St. Louis, MO, USA) and 10 μM monensin (Sigma-Aldrich Corp., St. Louis, MO, USA).

Supplementary figures

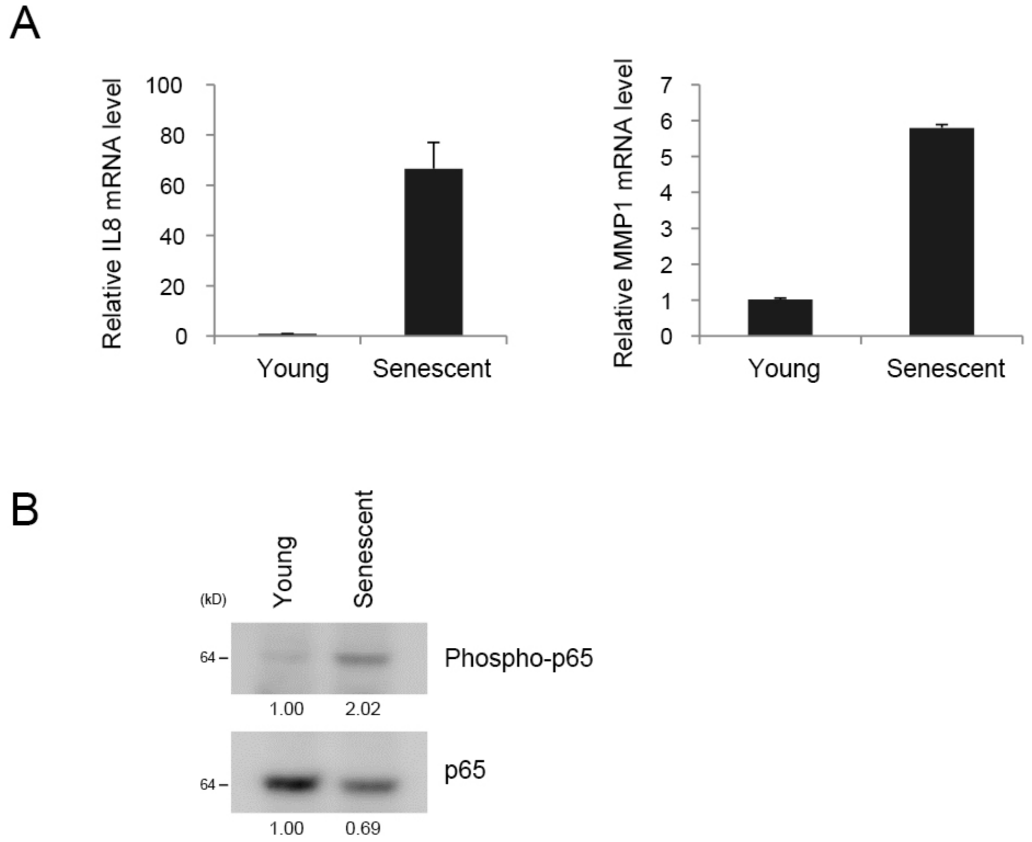

Supplementary Fig. S1: Expression of senescence-associated secretory phenotypes in human dermal fibroblasts. Young HDFs (PDL < 10) and senescent HDFs (PDL > 50) were analyzed for *IL-8* and *MMP1* mRNA expression (**a**) and phosphorylated (phosphor-p65) and total NF-κB (p65) protein levels (**b**) by qRT-PCR and western blot, respectively. The mRNA levels were normalized to that of *RPL13A*. GAPDH was the loading control for western blot. MMP1, matrix metalloproteinase 1.

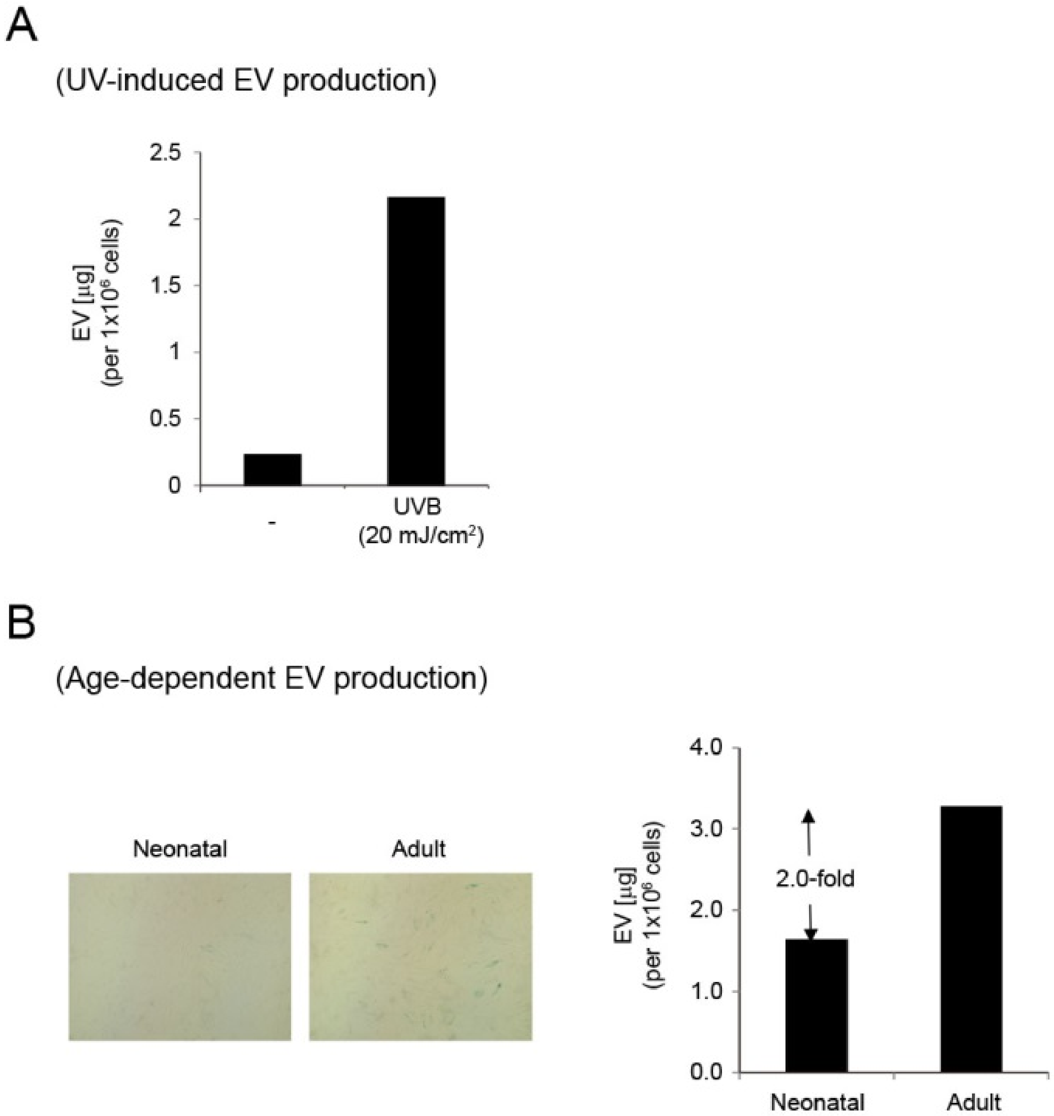

Supplementary Fig. S2: EV secretion was increased by UV irradiation and in aged fibroblasts. a HDFs (PDL < 10) were irradiated with UVB (20 mJ cm^{-2}). The numbers of EVs derived from equal numbers (1×10^6 cells) of UVB-treated or untreated HDFs were determined from a BCA protein assay. **b** Culture media for equal numbers (1×10^6 cells) of neonatal and adult (33-y) HDFs at the same passage (p16) were subjected to EV purification. Purified EV levels were determined by BCA protein assay. Representative data shown are fold differences between pairs of groups.

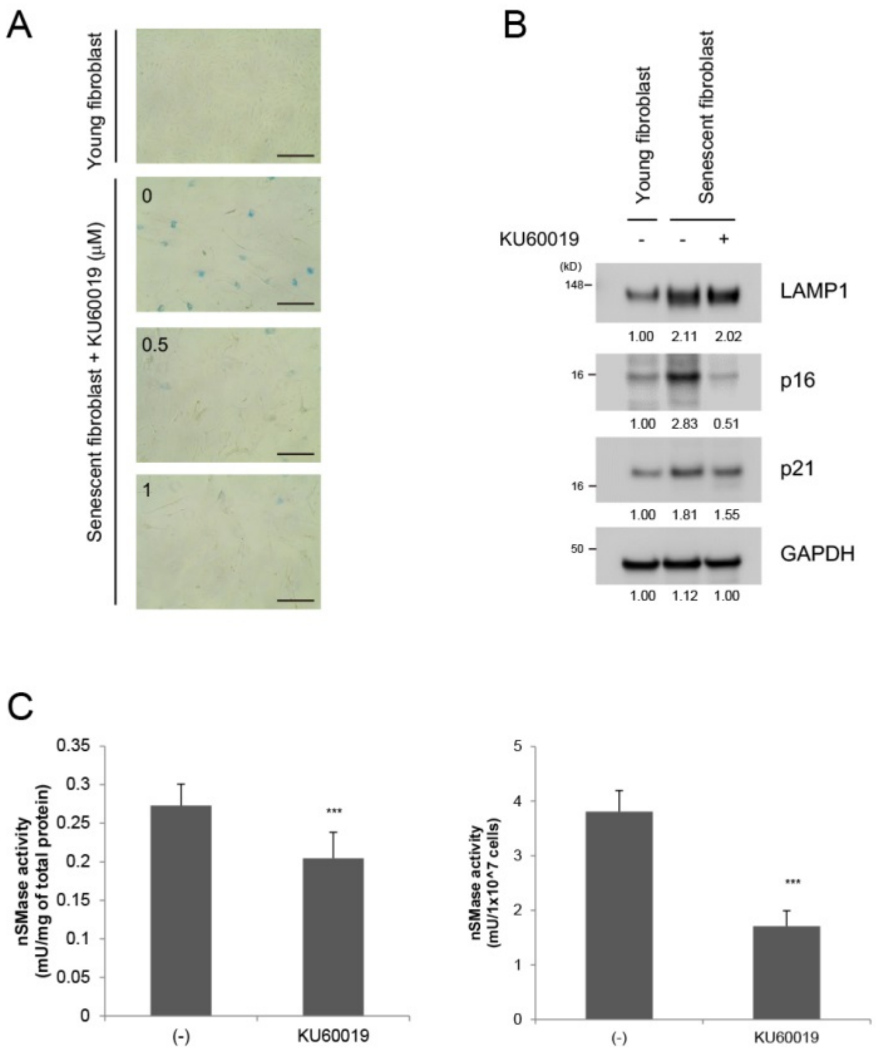

Supplementary Fig. S3: The effect of KU60019 in senescent HDFs. a Senescent HDFs (PDL > 50) were treated with various concentrations of KU60019 for 25 d and SA-β-gal staining in young (PDL < 10) or senescent HDFs was performed. Scale bar = 500 μm. **b**, **c** Senescent HDFs were treated with KU60019 (0.5 μM) for 25 d. **b** Western blot analyses for LAMP1, p16INK, and p21CIP were performed in young or senescent HDFs treated with or without KU60019. GAPDH, loading control. **c** nSMase activity was determined by the nSMase activity assay kit for equal amounts of protein or equal numbers of senescent HDFs. Data are means ± SD of three independent experiments using a senescent cell line (***$p < 0.001$).

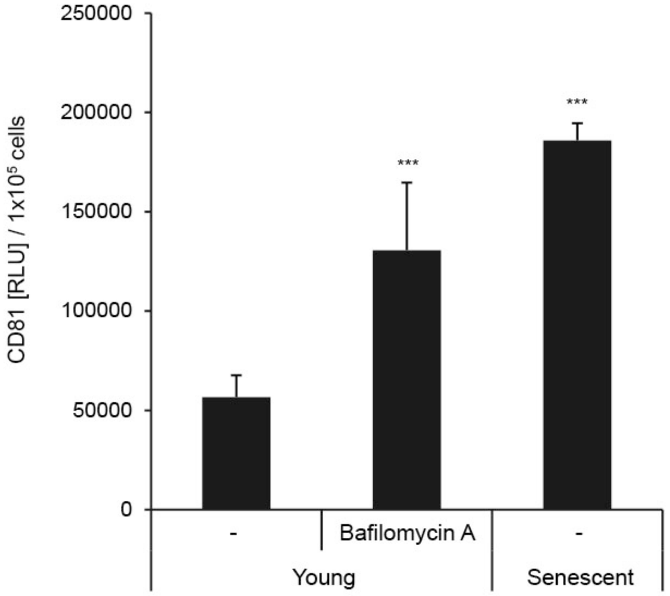

Supplementary Fig. S4: The effect of bafilomycin A on EV secretion. Young HDFs were treated with vehicle (-) or bafilomycin A (100 nM) for 24 h. The secreted EV levels from young HDFs with or without bafilomycin A or senescent HDFs were determined using a sandwich ELISA for CD81 form each conditioned medium. The data are expressed as the mean ± SD of three independent experiments (***p < 0.001).

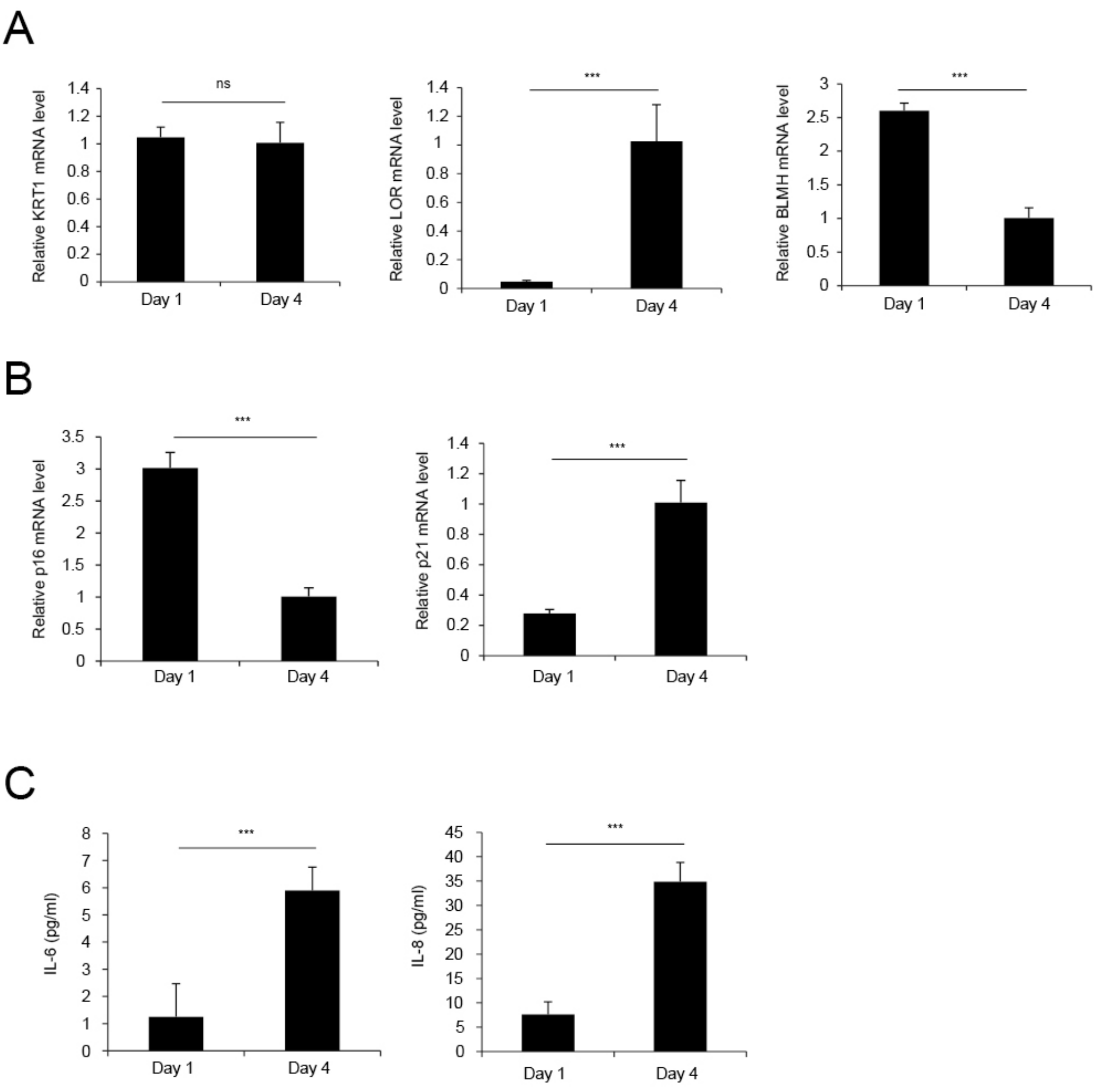

Supplementary Fig. S5: Expression of epidermal differentiation markers in human keratinocytes. HEKs were cultured for 1 d or 4 d. **a**, **b** Cells were analyzed daily by qRT-PCR for mRNA expression of keratinocyte differentiation-related (*KRT1*, *LOR*, *BLMH*) or cell cycle inhibition-related (*p16*, *p21*) markers. The mRNA levels were normalized to that of *RPL13A.* **c** Conditioned media were harvested at 1 d or 4 d. The IL-6 and IL-8 levels were measured with specific ELISA kits. Data are means ± SD of three independent experiments (***$p < 0.001$; ns, not significant).

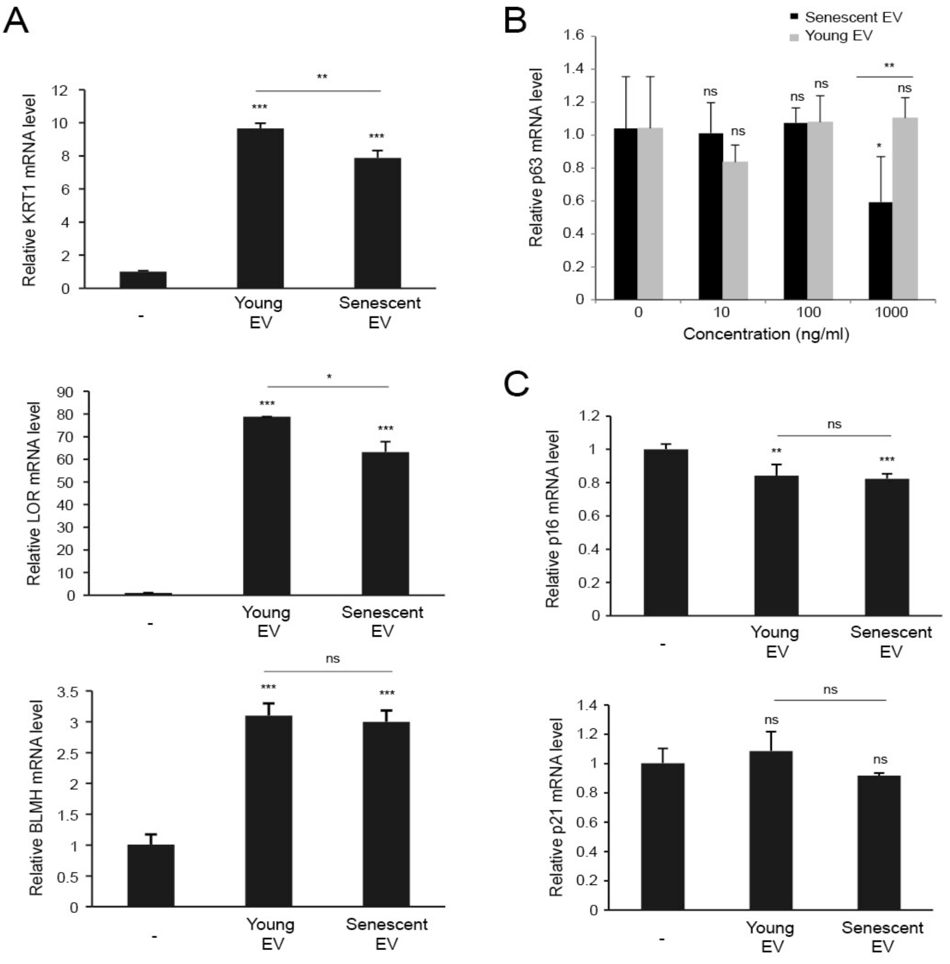

Supplementary Fig. S6: Comparison of the effects of young or senescent HDF-derived EVs on keratinocyte differentiation and senescence. a HEKs were treated with the same protein concentration (0.25 μg/ml) of young or senescent HDF-derived EVs for 4 d. The mRNA expression levels of *KRT1*, *LOR*, and *BLMH* were determined by qRT-PCR. Each mRNA level was normalized to that of *RPL13A*. b HEKs were treated for 4 d with various concentrations of the proteins from EVs derived from young or senescent HDFs. The *p63* mRNA expression levels were determined by qRT-PCR and normalized to that of *RPL13A*. c HEKs were treated with EVs derived from 5×10^5 cells of young or senescent HDFs for 4 d. The mRNA expression levels of *p16* and *p21* were measured using qRT-PCR. Each mRNA level was normalized to that of *RPL13A*. The data are expressed as the mean ± SD of three independent experiments (*$p < 0.05$; **$p < 0.01$; ***$p < 0.001$; ns, not significant).

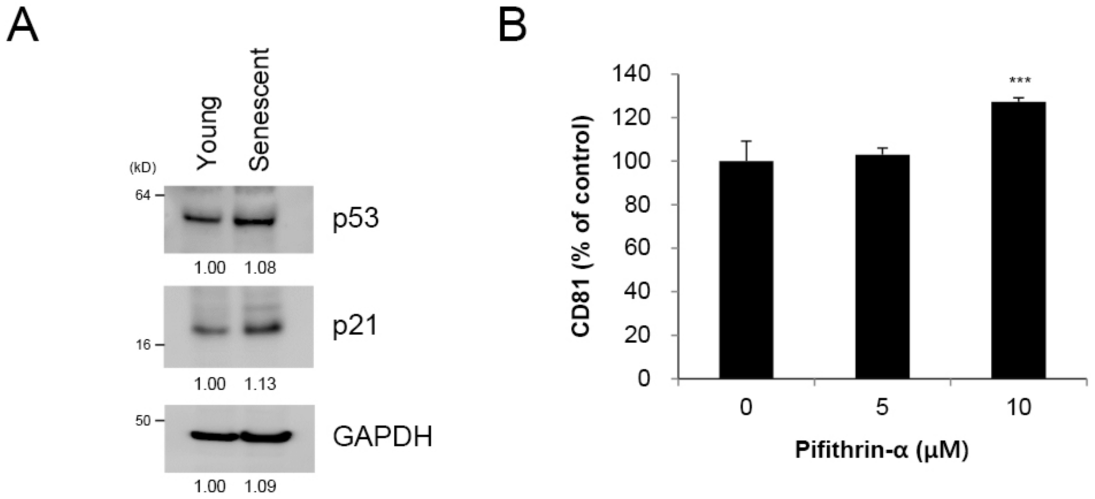

Supplementary Fig. S7: Effect of p53 on the increase in EV secretion in senescent HDFs. a Young (PD < 10) or senescent (PD > 50) HDFs were subjected to western blot using anti-p53 or anti-p21 antibodies. GAPDH, loading control. **b** Senescent HDFs were treated for 24 h with various concentrations of the p53 inhibitor pifithrin-α. EV levels were determined by sandwich ELISA for CD81. Data are means ± SD of three independent experiments using a senescent cell line (****p* < 0.001).

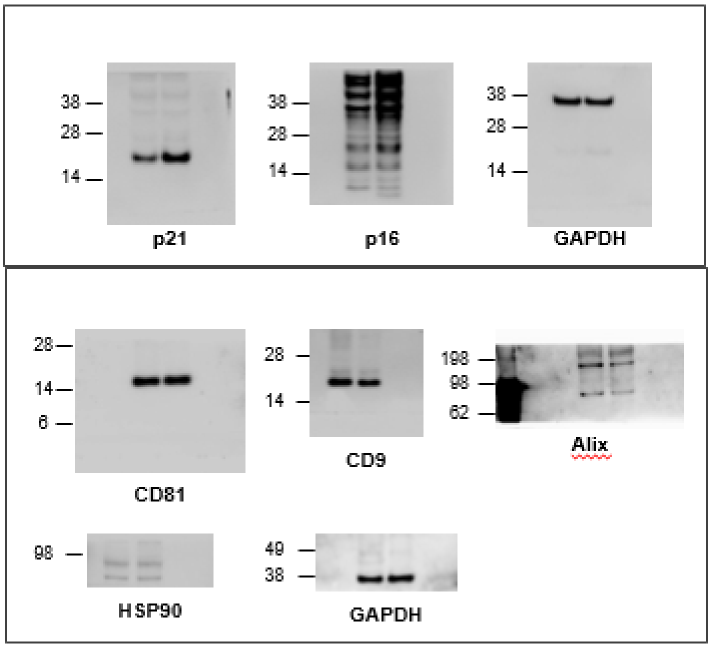

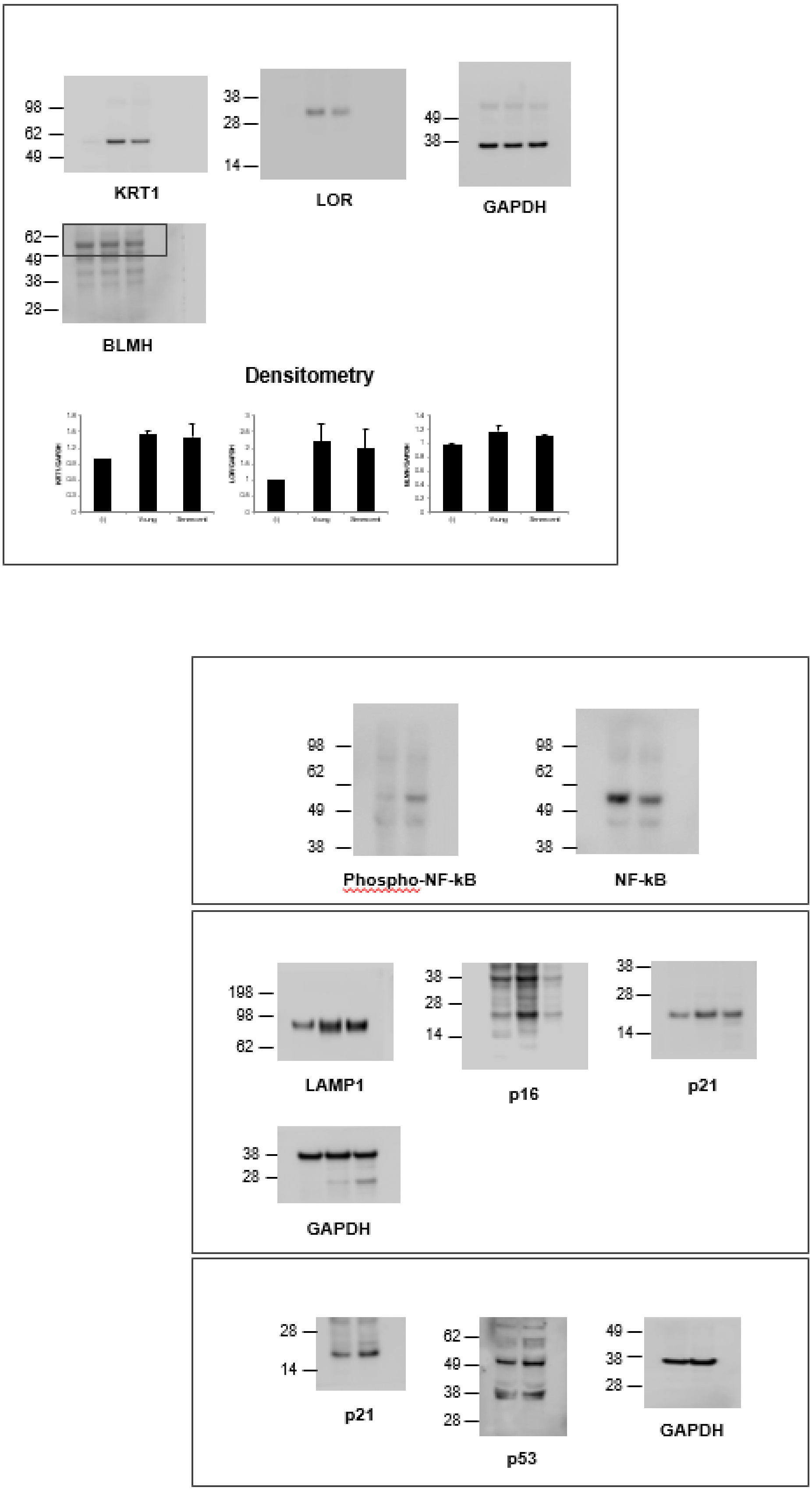

Supplementary Fig. S8: Full-length gels and blots images .

Supplementary reference

1 Kim, J.Y. *et al.* Pyruvate Protects against Cellular Senescence through the Control of Mitochondrial and Lysosomal Function in Dermal Fibroblasts. *J. Invest. Dermatol* **138**, 2522-2530(2018).
